# LRPPRC deficiency impairs trophoblast function through disrupted phosphatidylinositol metabolism in recurrent pregnancy loss

**DOI:** 10.1093/humrep/deag123

**Published:** 2026-07-28

**Authors:** Cancan Wang, Haoyu Yang, Mengyuan Li, Wenxuan Li, Xiafei Wu, Liping Jin, Xiang-Hong Xu

**Affiliations:** Shanghai Key Laboratory of Maternal Fetal Medicine, Clinical and Biological Sample Resource Center, Shanghai First Maternity and Infant Hospital, School of Medicine, Tongji University, Shanghai, P. R. China; Shanghai Key Laboratory of Maternal Fetal Medicine, Clinical and Biological Sample Resource Center, Shanghai First Maternity and Infant Hospital, School of Medicine, Tongji University, Shanghai, P. R. China; Shanghai Key Laboratory of Maternal Fetal Medicine, Clinical and Biological Sample Resource Center, Shanghai First Maternity and Infant Hospital, School of Medicine, Tongji University, Shanghai, P. R. China; Shanghai Key Laboratory of Maternal Fetal Medicine, Clinical and Biological Sample Resource Center, Shanghai First Maternity and Infant Hospital, School of Medicine, Tongji University, Shanghai, P. R. China; Shanghai Key Laboratory of Maternal Fetal Medicine, Clinical and Biological Sample Resource Center, Shanghai First Maternity and Infant Hospital, School of Medicine, Tongji University, Shanghai, P. R. China; Shanghai Key Laboratory of Maternal Fetal Medicine, Clinical and Biological Sample Resource Center, Shanghai First Maternity and Infant Hospital, School of Medicine, Tongji University, Shanghai, P. R. China; Shanghai Key Laboratory of Female Reproductive Endocrine Related Diseases, Hospital of Obstetrics and Gynecology, Shanghai Medical School, Fudan University, Shanghai, P. R. China; Shanghai Key Laboratory of Maternal Fetal Medicine, Clinical and Biological Sample Resource Center, Shanghai First Maternity and Infant Hospital, School of Medicine, Tongji University, Shanghai, P. R. China

**Keywords:** recurrent pregnancy loss, LRPPRC, trophoblast, phosphatidylinositol, metabolism, pregnancy outcome

## Abstract

**STUDY QUESTION:**

Is leucine-rich pentatricopeptide repeat-containing protein (LRPPRC) involved in the development of recurrent pregnancy loss (RPL) and, if so, what is the potential mechanism?

**SUMMARY ANSWER:**

LRPPRC deficiency impairs trophoblast viability and promotes apoptosis and oxidative stress through disruption of phosphatidylinositol (PI) metabolism, and low preconception serum PI (18:1/18:1) levels are associated with subsequent miscarriage in women with RPL.

**WHAT IS KNOWN ALREADY:**

RPL is commonly associated with impaired trophoblast function. LRPPRC works as a multifunctional protein that regulates a myriad of biological processes, including energy metabolism.

**STUDY DESIGN, SIZE, DURATION:**

The investigation included re-analysis of a public transcriptomic dataset, functional experiments in trophoblast cell models, lipidomic profiling of first-trimester human villous tissues obtained from 11 clinically healthy pregnant women and 11 patients with RPL, and analysis of preconception serum lipids in a cohort of 63 women with a history of RPL, who were prospectively followed for subsequent pregnancy outcomes resulting in 32 normal deliveries and 31 cases of early pregnancy loss.

**PARTICIPANTS/MATERIALS, SETTING, METHODS:**

Bioinformatics screening of the public transcriptomic dataset GSE76862 was first conducted to identify candidate genes dysregulated in RPL villous tissues. Following the identification of LRPPRC, its functional role was investigated through siRNA-mediated knockdown in the human trophoblast cells, specifically assessing the resultant effects on cell viability, apoptosis, and oxidative stress levels. To elucidate the downstream molecular pathways, transcriptomic analysis of the LRPPRC-depleted cells was performed. Subsequent Gene Ontology analyses revealed a significant enrichment of candidate differentially expressed genes that are involved in the phospholipid biosynthetic process. In parallel, comprehensive lipidomic profiling of human villous tissue samples from both RPL patients and matched controls was carried out using mass spectrometry to validate bioinformatic predictions at the metabolite level. To explore translational potential, the preconception serum levels of lipids, including PI species, were measured in a cohort of women with a history of RPL and statistically correlated with their subsequent pregnancy outcomes. Finally, direct functional rescue experiments were executed by exogenously supplementing PI (18:1/18:1) to the culture medium of LRPPRC-deficient trophoblast cells to determine if this intervention could reverse the observed phenotypic impairments.

**MAIN RESULTS AND THE ROLE OF CHANCE:**

LRPPRC was downregulated in RPL villous tissues. Functional validation in trophoblast cells demonstrated that knockdown of *LRPPRC* suppressed viability and increased apoptosis/oxidative stress. Transcriptomics and lipidomics converged on the phospholipid biosynthetic pathway as a key downstream process. Total lipids and specific phospholipids were decreased in RPL villous tissues. Low preconception serum PI (18:1/18:1) levels predicted subsequent miscarriage in the RPL cohort. Exogenous PI (18:1/18:1) supplementation reversed the cellular defects caused by LRPPRC deficiency.

**LARGE SCALE DATA:**

This study analyzed the publicly available transcriptomic dataset GSE76862 from the Gene Expression Omnibus (GEO) repository.

**LIMITATIONS AND REASONS FOR CAUTION:**

The analysis of preconception serum lipids was performed in a cohort of 63 women with a history of RPL. This sample size remains relatively limited, primarily due to the current availability of patients with documented follow-up pregnancy outcomes.

**WIDER IMPLICATIONS OF THE FINDINGS:**

Our results reveal a pivotal role for PI in the pathogenesis of RPL and highlight its potential as a mechanistic biomarker and therapeutic target.

**FUNDING:**

This study was supported by National Natural Science Foundation of China (82171657 and 81971384) and Shanghai First Maternity and Infant Hospital Basic Research Special Fund (2025A08).

**DISCLOSURES:**

The authors have declared no conflict of interest.

**TRIAL REGISTRATION NUMBER:**

N/A.

## Introduction

The process of pregnancy is characterized by intricate physiological interactions between the mother and the fetus, which hinge upon several critical factors, including trophoblast invasion, embryo quality, and receptivity of the endometrium. Trophoblast cells, which differentiate from the ectoderm of the blastocyst, play a pivotal role in initiating placentation and sculpting the immune micro-environment at the maternal–fetal interface (Liu *et al.*, 2022). During the first trimester, extravillous trophoblasts exhibit heightened proliferation and invasive capabilities, reminiscent of malignant tumor cells. However, under normal physiological conditions, this invasive activity is tightly regulated and diminishes following successful embryo implantation (Wang *et al.*, 2021). It is believed that abnormalities in extravillous trophoblasts may result in shallow placentation and incomplete formation of the maternal–fetal interface during the initial trimester, contributing significantly to the occurrence of recurrent pregnancy loss (RPL) (Romero *et al.*, 2011).

Lipid metabolism plays a crucial role in various biological processes, including cell proliferation, apoptosis, and stress response, and is particularly vital in trophoblast cells during early pregnancy (McKeegan and Sturmey, 2011; Jiang *et al.*, 2016; Ye *et al.*, 2021). Among the key lipid classes, phospholipids have long been recognized for their structural and signaling roles in placental and embryonic contexts (PORCELLATI *et al.*, 1959; Powell *et al.*, 2024). Notably, glycosylphosphatidylinositol (GPI) biosynthesis is essential for differentiation of the syncytiotrophoblast, a process critical for placental development (Alvarez-Sanchez *et al.*, 2024). Furthermore, some lipid-related enzymes such as stearoyl-CoA desaturase (SCD1) are reported to play a key role in *in vitro* blastocyst development and blastocyst implantation (Zhang *et al.*, 2024). Key enzymes in lipid synthesis, including SCD1, sterol regulatory element-binding protein 1c (SREBP1c), and acetyl-CoA carboxylase, are significant targets of RNA modifications (Liu *et al.*, 2024).

N6-methyladenosine (m^6^A) RNA methylation stands out as the most prevalent internal modification of mRNA. In most eukaryotic organisms, methylation within the 5′ untranslated region (UTR) of mRNA is crucial for mRNA splicing, editing, stability, degradation, and polyadenylation, whereas methylation in the 3′-UTR is associated with mRNA transport, translation initiation, and structural stability (Meyer *et al.*, 2012). Global m^6^A mapping of villous tissue has revealed a consistent hypomethylome in women with RPL (Li *et al.*, 2019;  Zhang *et al*., 2023b). However, the specific methyl-transferases, demethylases, or reader proteins that control this epitranscriptomic switch, and how their perturbation impairs extravillous trophoblast function, remain unclear.

This study identified leucine-rich pentatricopeptide repeat-containing protein (LRPPRC) as a differentially expressed m^6^A regulator in RPL through screening of the GSE76862 dataset from Gene Expression Omnibus. Beyond the observed statistical associations, LRPPRC presents a compelling mechanistic candidate for trophoblast dysfunction due to its established role as a master regulator of mitochondrial energy metabolism. LRPPRC is a nuclear-encoded RNA-binding protein that localizes primarily to mitochondria, where it binds directly to mitochondrial DNA-encoded mRNAs, stabilizing these transcripts and facilitating their translation into essential subunits of the oxidative phosphorylation (OXPHOS) complexes (Sasarman *et al.*, 2010; Xue *et al.*, 2025). Through this mechanism, LRPPRC serves as a critical coordinator of mitochondrial gene expression, ensuring proper assembly and function of the electron transport chain. However, deficiencies in LRPPRC lead to a marked decrease in mitochondrial mRNA levels, inhibition of mitochondrial protein synthesis, impaired assembly of complex IV, and reduced OXPHOS efficiency. These events ultimately trigger energy metabolic dysfunction and tissue-specific pathological phenotypes, including the French Canadian variant of Leigh syndrome (Sasarman *et al.*, 2010). Studies using human induced pluripotent stem cell models further confirm that downregulation of LRPPRC expression is consistent with reduced cytochrome c oxidase (COX) protein levels, and that tissues with high energy requirements are more profoundly affected (Gelinas *et al.*, 2024). Given that trophoblast invasion and placentation are highly energy-demanding processes that rely primarily on mitochondrial OXPHOS (Lehenauer *et al.*, 2025; Sah *et al.*, 2026), disruption of LRPPRC-mediated OXPHOS regulation would be predicted to compromise trophoblast bioenergetics, leading to functional deficits in migration and invasion, and ultimately resulting in aberrant placental development. Importantly, LRPPRC is expressed in human placental tissue at intermediate levels, supporting its functional relevance in this organ (Xu *et al.*, 2004). Thus, LRPPRC represents not merely a statistical correlate but a biologically plausible driver of trophoblast dysfunction. In this study, subsequent functional experiments in trophoblast cells demonstrated that siRNA-mediated LRPPRC silencing suppressed cell viability and concurrently induced apoptosis and oxidative stress. Transcriptomic analysis of LRPPRC-depleted cells further indicated significant enrichment of differentially expressed genes (DEGs) in the phospholipid biosynthetic process. Lipidomic profiling of villous tissues from RPL patients revealed a marked reduction in total lipids, including several phospholipid species. Moreover, we explored the prognostic potential of lipids and discovered that preconception serum levels of phosphatidylinositol (PI) (18:1/18:1) were significantly reduced in women with a history of RPL who subsequently experienced another miscarriage. Importantly, exogenous supplementation of PI (18:1/18:1) in LRPPRC-deficient cells effectively rescued the functional impairments in LRPPRC-deficient trophoblasts. Together, these results demonstrate a critical role for PI in RPL pathogenesis, likely through its influence on trophoblast function, and identify it as a promising predictive biomarker and potential therapeutic target.

## Materials and methods

### Data acquisition

The gene expression dataset GSE76862 associated with RPL was obtained from the Gene Expression Omnibus (GEO) database (https://www.ncbi.nlm.nih.gov/geo/info/datasets.html). This dataset comprises expression profiles from tissue samples of six individuals, including three healthy control (HC) subjects and three RPL patients. Based on a review of relevant publications, we manually curated a set of m^6^A modification regulators. The resulting gene list includes eight writers: Methyltransferase-like 3 (METTL3), Methyltransferase-like 14 (METTL14), RNA binding motif protein 15 (RBM15), RNA binding motif protein 15B (RBM15B), WTAP Wilms tumor 1-associating protein (WTAP), Vir-like m^6^A methyltransferase-associated protein (VIRMA), Cbl Proto-Oncogene Like 1 (CBLL1), and Zinc finger CCCH-type containing 13 (ZC3H13); 2 erasers: Fat mass and obesity-associated protein (FTO) and AlkB homolog H5 (ALKBH5) and 13 readers: YTH N6-methyladenosine RNA binding protein F1 (YTHDF1), YTH N6-methyladenosine RNA binding protein F2 (YTHDF2), YTH N6-methyladenosine RNA binding protein F3 (YTHDF3), YTH N6-methyladenosine RNA binding protein C1 (YTHDC1), YTH N6-methyladenosine RNA binding protein C2 (YTHDC2), IGF2BP1 Insulin-like growth factor 2 mRNA-binding proteins 1 (IGF2BP1), Insulin-like growth factor 2 mRNA-binding proteins 2 (IGF2BP2), Insulin-like growth factor 2 mRNA-binding proteins 3 (IGF2BP3), Heterogeneous nuclear ribonucleoprotein C (HNRNPC), Heterogeneous nuclear ribonucleoprotein A2/B1 (HNRNPA2B1), Fragile X mental retardation 1 (FMR1), ELAV like RNA binding protein 1 (ELAVL1), and LRPPRC.

### Screening of DEGs

Differential expression analysis between control and RPL samples was performed using the Limma package (Ritchie *et al.*, 2015), with the Benjamini–Hochberg procedure applied for multiple testing correction. Owing to the very limited number of DEGs identified after correction, an uncorrected threshold (*P *< 0.05, |log_2_FoldChange| > 1) was employed to maximize sensitivity. The results are summarized in a volcano plot. Subsequently, the list of candidate DEGs was cross-referenced with known m^6^A regulators, leading to the identification of an RPL-associated m^6^A candidate regulator that requires further validation.

### Clinical specimen collection

Preconception blood and the lipid metabolism-related indicators were collected from 313 RPL patients who attended the Department of Reproductive Immunology, Shanghai First Maternity and Infant Hospital from May 2022 to July 2024 prospectively. A total of 63 patients were followed up for the outcomes of subsequent pregnancies, among whom 32 had normal deliveries and 31 experienced early pregnancy loss. The descriptive statistics of the participants were shown in Table 1. First-trimester human villous tissues were collected from clinically healthy pregnant women and patients with recurrent early pregnancy loss. The inclusion criteria for RPL comprised women of reproductive age with a history of two or more spontaneous pregnancy losses. Any verifiable cause, including chromosomal abnormalities, anatomic deformation, infection, endocrine or metabolic diseases, or autoimmune response, was excluded. The characteristics of villous tissue donors were listed in Supplementary Table S1.

**Table 1.** Descriptive statistics of the followed-up participants at baseline.

Variables	Total subjects (n = 63)	Followed up		*P*-value^c^
		Failure^a^ (n = 31)	Success^b^ (n = 32)	
Age (years) (mean ± SD)	30.95 ± 3.35	30.68 ± 3.28	31.22 ± 3.44	0.525
BMI (kg/m^2^) (mean ± SD)	21.81 ± 3.00	22.25 ± 3.43	21.38 ± 2.48	0.252
Total cholesterol (mmol/l) (mean ± SD)	5.01 ± 1.08	5.23 ± 1.06	4.88 ± 1.10	0.303
Apolipoprotein A1 (g/l) (mean ± SD)	1.91 ± 0.53	1.73 ± 0.61	2.04 ± 0.45	0.240
Apolipoprotein B (g/l) (mean ± SD)	0.98 ± 0.22	1.00 ± 0.21	0.95 ± 0.24	0.670
A1/B (mean ± SD)	2.01 ± 0.57	1.73 ± 0.54	2.21 ± 0.53	0.087
High-density lipoprotein (mmol/l) (mean ± SD)	1.93 ± 0.54	1.83 ± 0.59	1.99 ± 0.53	0.596
Low-density lipoprotein (mmol/l) (mean ± SD)	2.94 ± 0.94	3.19 ± 0.73	2.79 ± 1.05	0.432
Triglycerid (mmol/l) (median (q_1_, q_3_))	1.17 (0.78, 2.05)	1.98 (1.13, 2.43)	1.09 (0.69, 1.95)	**0.033** ^d^
Number of prior spontaneous pregnancy losses (median (q_1_, q_3_))	2.00 (2.00, 3.00)	3.00 (2.00, 3.00)	2.00 (2.00, 3.00)	**0.035** ^d^

a Failure group included patients with recurrent pregnancy loss whose subsequent pregnancy again resulted in miscarriage.

b Success group included patients with recurrent pregnancy loss who achieved a successful subsequent pregnancy.

c *P*-value was calculated by two-tailed unpaired Student’s *t*-test for normally distributed data (age, BMI, total cholesterol, Apolipoprotein A1, Apolipoprotein B, A1/B, high-density lipoprotein and low-density lipoprotein) or two-tailed Mann–Whitney test for non-normally distributed data (triglyceride, number of prior spontaneous pregnancy losses).

d Bold values indicate statistical significance at the threshold of *P* < 0.05.

q_1_, first quartile; q_3_, third quartile.

After curettage, the tissues were immediately collected under sterile conditions into pre-chilled PBS. The samples were then either used for trophoblast isolation or processed for gene expression and localization analysis. For mRNA and protein extraction, samples were frozen in liquid nitrogen and stored in the refrigerator at −80°C until use. The remaining tissues were fixed at 4°C using 4% paraformaldehyde and embedded in paraffin for histological analysis. Blood was collected in clot tubes and allowed to coagulate by leaving it undisturbed at room temperature for 30 min, followed by centrifugation. Serum was then isolated and stored at −80°C. This study was approved by the Scientific and Ethics Committee of Shanghai First Maternity and Infant Hospital (Approval No. 2021(06), 2023(040), and 2026(138)). Written informed consent was obtained from all patients before sampling.

### Real-time quantitative polymerase chain reaction

Total RNA from human villous tissues was prepared with TransZol (ET101-01, Trans Gen Biotech). The reverse transcriptase kit (No. RR037A, TaKaRa) is used to reverse total RNA into cDNA. Real-time quantitative polymerase chain reaction (RT–qPCR) was performed using a QuantStudio 5 analyzer (Applied Biosystems, Carlsbad, CA, USA) with TB Green Premix Ex Taq™ II (No. RR820A, Takara) following the manufacturer’s instructions. All gene expressions are normalized based on the β-actin mRNA levels in each sample using the 2^ − ΔΔCt^ method. The primer sequences are listed in Supplementary Table S2.

### Western-blot analysis

Lysates were prepared using RIPA lysis buffer (No. WB3100, NCMbio) supplemented with a protease inhibitor cocktail (No. P001, NCMbio). Proteins were separated by SDS–polyacrylamide gel electrophoresis and transferred to a PVDF membrane. The membrane was blocked with 5% non-fat milk for 2 h at room temperature and incubated with antibodies against LRPPRC (1:10 000, No. 21175-1, Proteintech), Caspase-3 (1:500, No. 14220, CST), Cleaved Caspase-3 (1:500, No. 9664, CST), Caspase-9 (1:500, No. 9508, CST), Cleaved Caspase-9 (1:500, No. 52873, CST), cleaved PARP (1:500, No. F0136, Selleck), Cytochrome c (1:1000, No. F2534, Selleck), COX IV (1:1000, No. WL02203, Wanleibio), Tublin (1:1000, No. M20005, Abmart), and β-actin (1:1000, No. 4970S, CST) at 4°C overnight. Then, the blots were incubated for 1 h at room temperature with an HRP-conjugated secondary antibody. The immunoreactive bands were detected with an enhanced chemiluminescence solution (No. 180-5001, Tanon) and then visualized using a FluorChem E imaging instrument. Then, ImageJ was applied to quantify the relative protein levels.

### Histological analysis

The location of LRPPRC was detected using immunohistochemistry assays with antibodies against LRPPRC (1:250, No. 21175-1, Proteintech). The experiments were repeated with at least three different samples from each group. Image acquisition was performed using a Pannoramic 250 Flash digital microscope (3DHISTECH, Budapest, Hungary).

### Cell culture and treatment

HTR-8/SVneo cells were cultured in DMEM/F-12 supplemented with 10% fetal bovine serum and 1% penicillin/streptomycin at 37°C in humidified air containing 5% CO_2_. Primary human trophoblasts were isolated from fresh villous tissues of HC subjects. Tissues were rinsed with PBS, minced into a paste, and evenly placed onto the bottom of a culture flask pre-wetted with trophoblast medium. The flask was inverted and incubated for 4 h, after which complete medium was added. The culture was maintained with medium changes every 3 days. After reaching confluence, fibroblast-like cells were removed by differential digestion, and the remaining epithelial-like cells were used as primary trophoblasts, with a purity of > 90% as assessed by cytokeratin-7 (CK7) staining (Fig. 1D). To evaluate the roles of LRPPRC, cells were transfected with siRNA. The sequences of the siRNAs used were listed in Supplementary Table S2.

**Figure 1.**
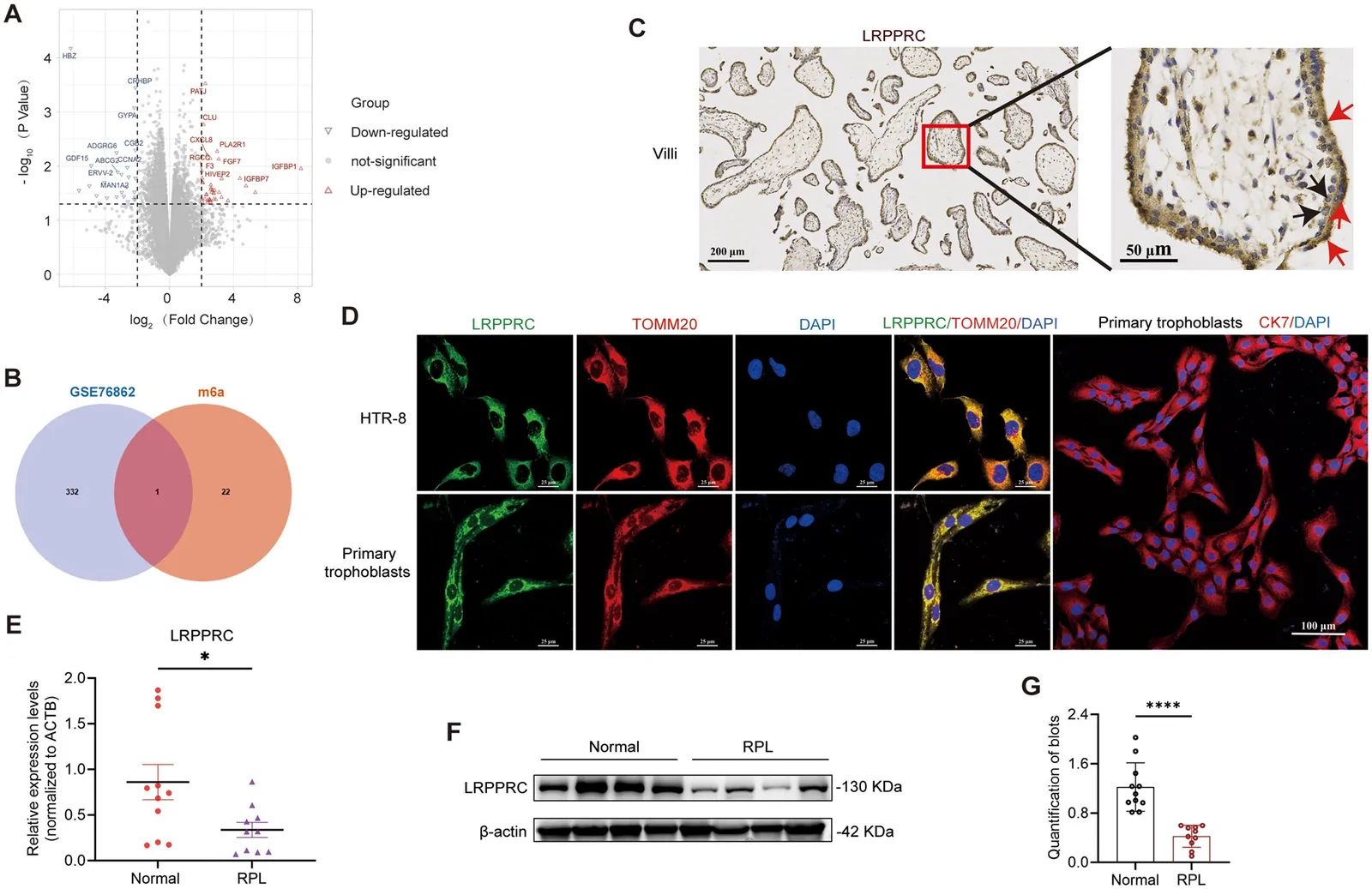
**LRPPRC expression was downregulated in RPL.** (**A**) Volcano plot displaying candidate DEGs between RPL patients and healthy controls from the GSE76862 dataset. (**B**) Venn diagram identifying LRPPRC as the only candidate disregulated m^6^A regulator among the DEGs. (**C**) Representative images of expression and localization of LRPPRC in first trimester villi by immunohistochemistry analysis. Brownish color represents positive staining. Black arrow: cytotrophoblasts, red arrow: syncytiotrophoblasts. (**D**) Representative images of LRPPRC expression and localization in HTR-8/SVneo cells and primary trophoblast cells, co-stained with the mitochondrial marker TOMM20. Primary trophoblasts exhibited > 90% purity, as determined by CK7 immunostaining. (**E**) RT–qPCR analysis of LRPPRC mRNA levels in placental villi from normal control subjects and RPL patients. (**F**) Representative western-blot images of LRPPRC protein levels in placental villi from normal control subjects and RPL patients, with β-actin serving as a loading control. (**G**) Quantification of LRPPRC protein grayscale values from (E). Data are presented as mean ± SD. *P < 0.05, ****P < 0.0001. LRPPRC, leucine-rich pentatricopeptide repeat-containing protein; RPL, recurrent pregnancy loss; DEGs, differentially expressed genes; TOMM20, translocase of outer mitochondrial membrane 20; CK7, cytokeratin 7; RT–qPCR, real-time quantitative polymerase chain reaction.

PI (18:1/18:1) (No. 850149P-500UG, Merck) was suspended in DMEM containing 10% FCS, vigorously mixed, and sonicated at room temperature for 20 min to form phospholipid vesicles, which were then supplemented to the cell culture medium. When combined with siRNA treatment, medium containing siRNA/lipofectamine RNAiMax complex was replaced with complete medium for 5 h, and then changed to phospholipid-containing medium.

### Immunofluorescence microscopy

Cells were seeded in glass bottom cell culture dishes. Then, the cells were fixed with 4% paraformaldehyde for 30 min and permeabilized with 0.3% Triton X-100 for 5 min at room temperature. After washing with PBS and blocking by incubation in normal goat serum, the cells were incubated overnight with primary antibodies against CK7 (1:100, No. A4765, ABclonal), LRPPRC (1:200, No. 21175-1, Proteintech), or TOMM20 (1:100, No. ET1609-25, Huabio). Following washing, the cells were incubated with Polymer-HRP secondary antibodies for 30 min, followed by TYR fluorescent dyes (No. AFIHC034, AiFang biological) for 5–10 min. Nuclei were stained with DAPI (No. G1407, Servicebio). Fluorescence images were acquired using a confocal microscope.

### CCK-8 and cell-ATP assays

The viabilities of cells were assessed using Cell Counting Kit-8 reagent and CTG Cell Viability Detection Reagent (No. HY-K0314, MedChemExpress) according to the manufacturer’s instructions.

### Cytotoxicity analysis

Cytotoxicity was measured using a lactate dehydrogenase (LDH)-Cytotoxicity Assay Kit (No. HY-K1090, MedChemExpress) according to the manufacturer’s instructions on Multiskan FC tablet reader.

### Cell apoptosis analysis

A total of 1 × 10^6^ cells were resuspended in 100 μl of 1× binding buffer, stained with 5 μl Annexin V-FITC and 5 μl of propidium iodide (No. RE204101, Biotend), and incubated for 15 min in the dark at 25°C. After the addition of 400 μl 1× binding buffer, samples were immediately analyzed on a flow cytometry. Apoptotic distribution was quantified with FlowJo_V10 software.

### Cytochrome c release assay

Mitochondria-depleted cytosolic proteins were prepared using the Cell Mitochondria Isolation Kit (No. C3601, Beyotime) to evaluate cytochrome c release into the cytoplasm by western blotting. Successful depletion of mitochondrial residues from the cytosol was verified via western blotting for the mitochondrial marker COX IV.

### Targeted lipidomics absolute quantitative analysis

The experiment and data analysis were supported by Shanghai Zhongke New Life Technology Co., Ltd. MS-grade methanol (A452-4-CASE), MS-grade acetonitrile (271004), and HPLC-grade 2-propanol (A461-4-CASE) were purchased from Thermo Fisher. HPLC-grade formic acid (33015) and HPLC-grade ammonium formate (70221-100G-F) were purchased from Sigma–Aldrich.

#### Sample preparation and lipid extraction

Two hundred microliters of water was added to the sample and vortexed for 5 s, then 240 μl of pre-cooled methanol was added, and the mixture was vortexed for 30 s. After that, 800 μl of methyl tert-butyl ether was added, sonicated at 4°C for 20 min, and then allowed to stand at room temperature for 30 min. The solution was centrifuged at 14 000*g* at 10°C for 15 min, and the upper organic solvent layer was obtained and dried under nitrogen.

#### LC-MS/MS lipid analysis and detection

Reversed-phase chromatography was performed using a CSH C18 column (1.7 μm, 2.1 mm × 100 mm, Waters) for LC separation. The lipid extract was re-dissolved in 200 μl of 90% isopropanol/acetonitrile, centrifuged at 14 000*g* for 15 min, and finally 3 μl of the sample was injected. Solvent A was acetonitrile-water (6:4, v/v) containing 0.1% formic acid and 0.1 mM ammonium formate, and solvent B was acetonitrile-isopropanol (1:9, v/v) containing 0.1% formic acid and 0.1 mM ammonium formate. The initial mobile phase was 30% solvent B with a flow rate of 300 μl/min. Hold for 2 min, then linearly increase to 100% solvent B within 23 min, then equilibrate in 5% solvent B for 10 min. Mass spectra in positive and negative modes were obtained with Q-Exactive Plus, respectively. The electrospray ionization parameters were optimized and set as follows: source temperature 300°C, capillary temperature 350°C, ion spray voltage 3000 V, lens radio frequency level 50%, and instrument scanning range 200–1800 m/z.

LipidSearch was used for peak identification, peak extraction, lipid identification (secondary identification) of lipid molecules, and internal standard lipid molecules. The main parameters were: precursor tolerance: 5 ppm, product tolerance: 5 ppm, product ion threshold: 5%. The extracted data were first evaluated for quality, and then analyzed after passing.

Partial least squares discrimination analysis is a supervised discriminant analysis statistical method. This method uses partial least squares regression to establish a relationship model between lipid expression and sample categories to realize the prediction of sample categories. Through the established discriminant model, differential lipids related to grouping can be screened from the dataset. The variable important in projection (VIP) method of model variables and the univariate statistical analysis *T*-test *P*-value were used. First, differential metabolites were screened by VIP ≥ 1, and then further screened by *T*-test *P *< 0.05 to finally find differential metabolites.

LIPID MAPS (https://www.lipidmaps.org/), Human Metabolome Database, and Kyoto Encyclopedia of Genes and Genomes data sources and the plug-in LipidBlast database were selected for the identification of lipid metabolites. Enrichment analysis was performed through the online database Metascape (http://www.metaboanalyst.ca).

### Statistical analysis

Data were expressed as means ± SD or medians. Statistical analyses were performed using SPSS version 22.0, GraphPad Prism version 10.1.2, and R version 4.3.0. Experiments were performed in triplicate and conducted at least three independent times to ensure data reliability. Differences with *P *< 0.05 were considered statistically significant. For all statistical analyses, asterisks representing *P* are indicated in each figure legend, with ns indicating ‘not significant’ (*P *> 0.05).

## Results

### LRPPRC was downregulated in the RPL patients

In this study, we conducted an exploratory analysis of the publicly available transcriptomic dataset GSE76862 to compare gene expression profiles between patients with RPL and HCs. Owing to the very limited number of DEGs identified after Benjamini–Hochberg multiple testing correction, an uncorrected threshold of *P *< 0.05 and |log_2_FoldChange| > 1 was employed to maximize sensitivity, yielding 333 candidate DEGs (Fig. 1A). To explore the potential role of m^6^A RNA methylation regulators in the pathogenesis of RPL, we cross-referenced the DEGs with 23 known m^6^A regulators obtained from the literature (Li *et al.*, 2023). Among the 23 regulators, LRPPRC was the only overlapping gene, suggesting it as a candidate m^6^A regulatory gene associated with RPL (Fig. 1B). We also noted that several other m^6^A regulators (e.g. HNRNPC and VIRMA), though not meeting the screening criteria, exhibited a modest decrease in expression (Supplementary Table S3). These observations warrant further validation.

To dissect the underlying function of LRPPRC in the placenta, we first determined its localization in first-trimester villi using immunohistochemistry. LRPPRC was robustly expressed in the cytoplasm of syncytiotrophoblasts and cytotrophoblasts (Fig. 1C). We further examined the subcellular localization of LRPPRC in primary human trophoblasts and HTR-8/SVneo cells and we found that LRPPRC was colocalize with mitochondria in both cell types (Fig. 1D). Next, we compared LRPPRC expression in placental villi between normal controls and RPL patients. qPCR and western blotting revealed that both mRNA and protein levels of LRPPRC were markedly downregulated in the RPL group (Fig. 1E–G).

### LRPPRC downregulation impaired trophoblast function

Given the predominant localization of LRPPRC in first-trimester trophoblasts and its significant downregulation in RPL placental tissues, we hypothesized that LRPPRC loss of function contributes to RPL pathogenesis. To test this, we knocked down LRPPRC in the human trophoblast cell line HTR-8/SVneo using siRNA. LRPPRC depletion led to a marked impairment in cell proliferation (Fig. 2A and B) and viability (Fig. 2C). Consistent with this, LDH release assay and flow cytometry confirmed a significant increase in apoptosis upon *LRPPRC* knockdown (Fig. 2D–F). This pro-apoptotic effect was further supported by western-blot analysis, which showed elevated levels of key apoptotic marker proteins (Fig. 2G). To investigate the underlying mechanism, we examined mitochondrial pathway activation. Cytosolic protein extraction revealed that *LRPPRC* knockdown led to increased cytochrome c levels in the cytosol (Fig. 2G), indicating mitochondrial outer membrane permeabilization and directly linking LRPPRC’s mitochondrial function to the activation of the intrinsic apoptotic pathway. To explore the potential upstream triggers, we assessed cellular stress and found that *LRPPRC* silencing increased reactive oxygen species (ROS) levels (Fig. 2H and I). Importantly, the impact of *LRPPRC* knockdown on cell viability and apoptosis was further validated in primary trophoblast cells (Fig. 3), consistent with our findings in the HTR-8/SVneo cell line. Collectively, these results demonstrate that LRPPRC deficiency enhances cellular stress and apoptosis while attenuating cell viability in trophoblasts.

**Figure 2.**
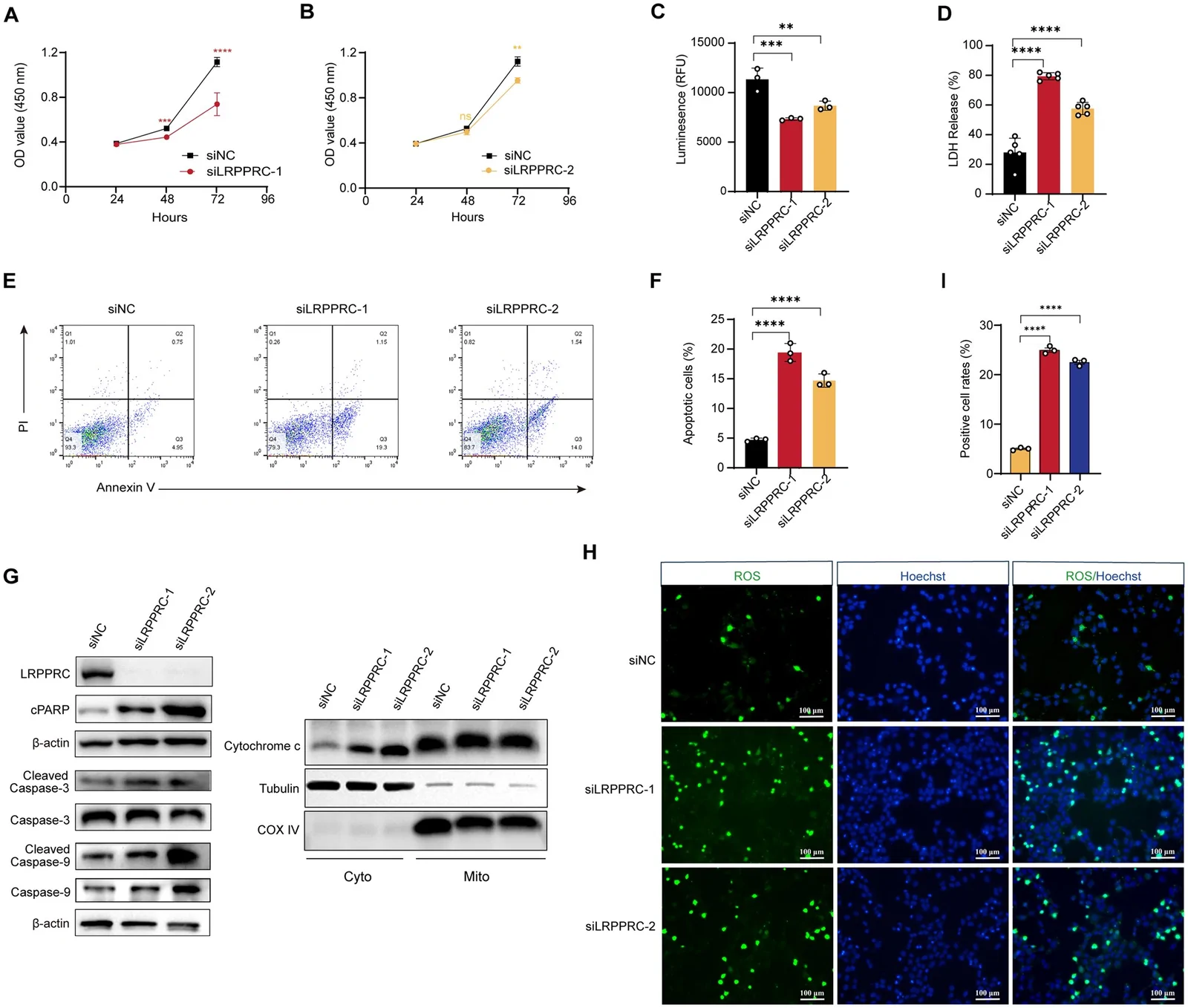
**LRPPRC downregulation impaired HTR-8/SVneo cell function.** (**A and B**) Cell proliferation assessed by CCK-8 assay in HTR-8/SVneo cells following *LRPPRC* knockdown. (**C**) Cell viability measured by ATP assay. (**D**) Cell cytotoxicity determined by LDH release assay. (**E**) Apoptosis analysis performed by Annexin V/Propidium Iodide double staining followed by flow cytometry. (**F**) Quantification of apoptotic cell rates. (**G**) Western-blot analysis of caspase-3, caspase-9, and cPARP expression in siLRPPRC-treated HTR-8/SVneo cells; β-actin served as a loading control. To evaluate cytochrome c release into the cytoplasm, mitochondria-depleted cytosolic fractions were prepared, with tubulin serving as a loading control. Efficient removal of mitochondrial material from the cytosolic fraction was confirmed by the loss of the mitochondrial marker COX IV. (**H**) Representative fluorescence microscopy images of cells stained with DCFH-DA for ROS detection. Scale bar, 100 μm. (**I**) Quantitative analysis of positive cells from (H). Data are presented as mean ± SD. *P < 0.05, **P < 0.01, ***P < 0.001, ****P < 0.0001. LRPPRC, leucine-rich pentatricopeptide repeat-containing protein; CCK-8, Cell Counting Kit-8; ATP, adenosine 5′-triphosphate; LDH: lactate dehydrogenase; cPARP, cleaved poly (ADP-ribose) polymerase; COX IV, cytochrome c oxidase IV; DCFH-DA, 2′,7′-dichlorodihydrofluorescein diacetate; ROS, reactive oxygen species; OD, optical density; ns, not significant; RFU, relative fluorescence units; siNC, negative control siRNA; cyto, cytosolic; mito, mitochondrial.

**Figure 3.**
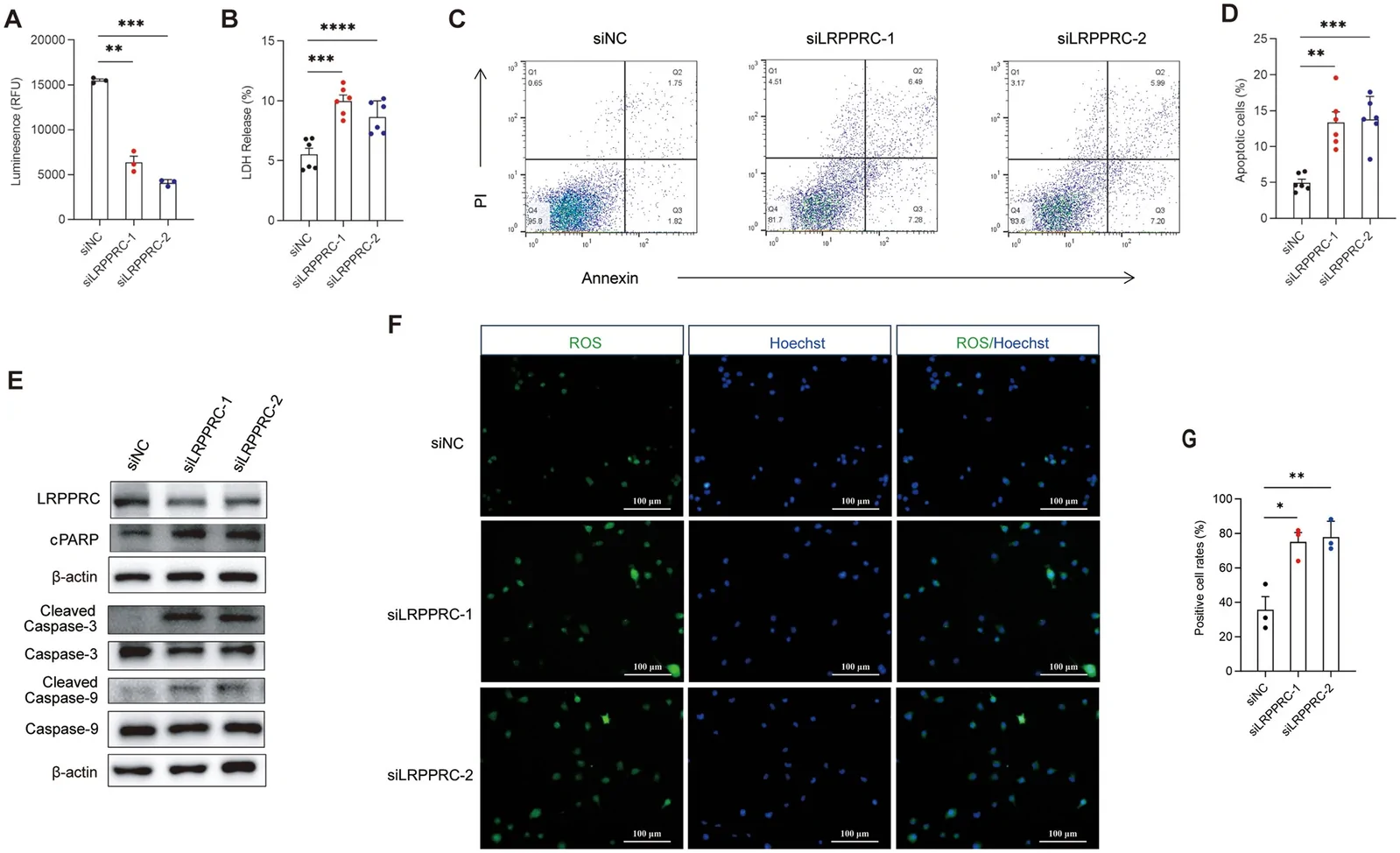
**LRPPRC downregulation impaired primary human trophoblast function.** (**A**) Cell viability measured by ATP assay. (**B**) Cell cytotoxicity determined by LDH release assay. (**C**) Apoptosis analysis performed by Annexin V/Propidium Iodide double staining followed by flow cytometry. (**D**) Quantification of apoptotic cell rates. (**E**) Western-blot analysis of caspase-3, caspase-9, and cPARP expression in siLRPPRC-treated primary trophoblast cells; β-actin served as a loading control. (**F**) Representative fluorescence microscopy images of cells stained with DCFH-DA for ROS detection. Scale bar, 100 μm. (**G**) Quantitative analysis of positive cells from (**F**). Data are presented as mean ± SD. *P < 0.05, **P < 0.01, ***P < 0.001, ****P < 0.0001. LRPPRC, leucine-rich pentatricopeptide repeat-containing protein; ATP, adenosine 5′-triphosphate; LDH, lactate dehydrogenase; cPARP, cleaved poly (ADP-ribose) polymerase; DCFH-DA, 2′,7′-dichlorodihydrofluorescein diacetate; ROS, reactive oxygen species; RFU, relative fluorescence units; siNC, negative control siRNA.

### Abnormal lipid metabolism in *LRPPRC* knockdown trophoblasts and RPL patients

To explore the mechanistic basis of LRPPRC in RPL, we carried out transcriptome sequencing in trophoblast cells following *LRPPRC* knockdown. Differential expression analysis (|log_2_FoldChange| > 1, *P *< 0.05) and subsequent GO enrichment implicated the phospholipid biosynthetic pathway as a key process affected by LRPPRC deficiency (Fig. 4A), suggesting a potential link between LRPPRC and lipid metabolism. Prompted by this finding, we performed lipidomic profiling on clinical villous samples. Consistent with the above gene expression data, RPL tissues exhibited a marked decrease in total lipids (Fig. 4B), driven largely by a reduction in several phospholipid classes (Fig. 4C).

**Figure 4.**
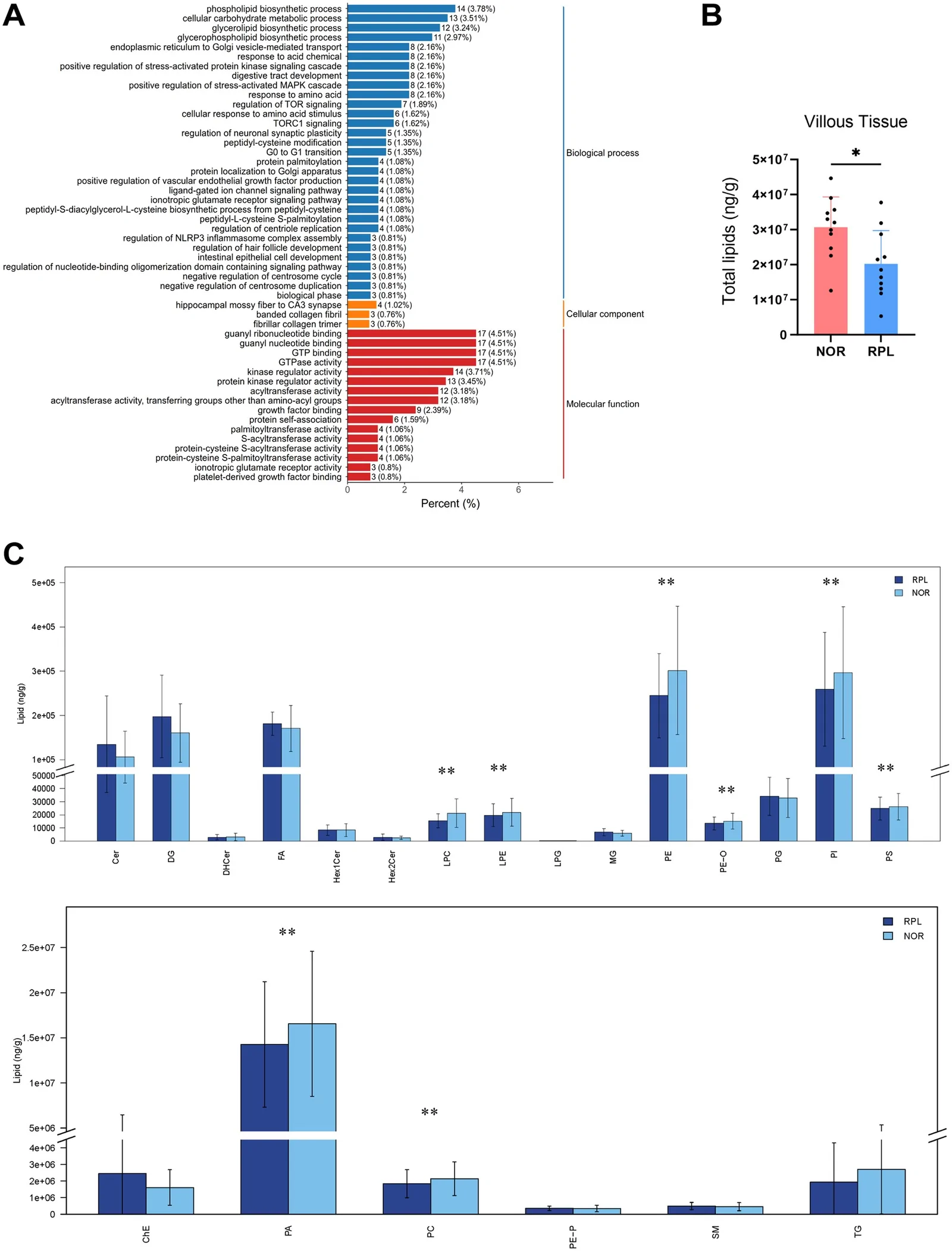
**Transcriptomic and lipidomic analyses linked LRPPRC deficiency to disrupted phospholipid metabolism.** (**A**) GO enrichment analysis of differentially expressed genes in trophoblasts after *LRPPRC* knockdown shows significant enrichment for the phospholipid biosynthetic process. (**B**) Total lipid levels are significantly reduced in villous tissues from RPL patients (n = 11) compared to healthy controls (NOR, n = 11). (**C**) Comparison of major lipid class abundances between RPL and NOR villous tissues. *P < 0.05, **P < 0.01. LRPPRC, leucine-rich pentatricopeptide repeat-containing protein; GO, gene ontology; RPL, recurrent pregnancy loss; NOR, normal; Cer, Ceramides; DG, diglyceride; DHCer, dihydroceramides; FA, fatty acid; Hex1Cer, hexosylceramide; Hex2Cer, dihexosylceramide; LPC, lysophosphatidylcholine; LPG, lysophosphatidylglycerol; LPE, lysophosphatidylethanolamine; MG, monoglyceride; PG, phosphatidylglycerol; PE, phosphatidylethanolamine; PE-O, phosphatidylethanolamine O-alkyl ether; PI, phosphatidylinositol; PS, phosphatidylserine; ChE, Cholesterol Ester; PA, phosphatidic acid; PC, phosphatidylcholine; PE-P, phosphatidylethanolamine plasmalogen; SM, sphingomyelin; TG, triglyceride.

To further assess the prognostic potential of these lipid signatures, we analyzed pre-gestational serum samples from 63 women with a history of RPL and tracked their subsequent pregnancy outcomes (categorized as success or failure) (Table 1). While no significant differences were observed in total lipid levels between the two outcome groups (Fig. 5A), a comparison of lipid subtypes identified six specific lipids that were significantly altered (Fig. 5B). We then evaluated the predictive power of each of the six lipids for subsequent pregnancy outcome by calculating their area under the curve (AUC) values. The data indicate that five of the six lipids (all except TG (14:0_18:2_20:5)) exhibited modest discriminatory ability, with individual AUCs ranging from 0.635 to 0.693 (*P*-value <0.1) (Supplementary Table S4). In addition, to address potential confounding effects, we also evaluated the predictive performance of three potential covariates (maternal age, pre-pregnancy BMI, and number of prior spontaneous pregnancy losses) using univariate ROC analysis. Among these, the number of prior spontaneous pregnancy losses showed marginal significance was therefore used as an adjusting covariate. Based on this criterion, five lipids (those with *P *< 0.10) together with the marginal significant covariate (number of prior spontaneous pregnancy losses) were entered into the final logistic regression model. Using this refined approach, the combined multivariable model achieved an AUC of 0.859 (Supplementary Table S4, Fig. 5C), demonstrating substantially improved discriminative ability after accounting for both lipid profiles and an important confounder.

**Figure 5.**
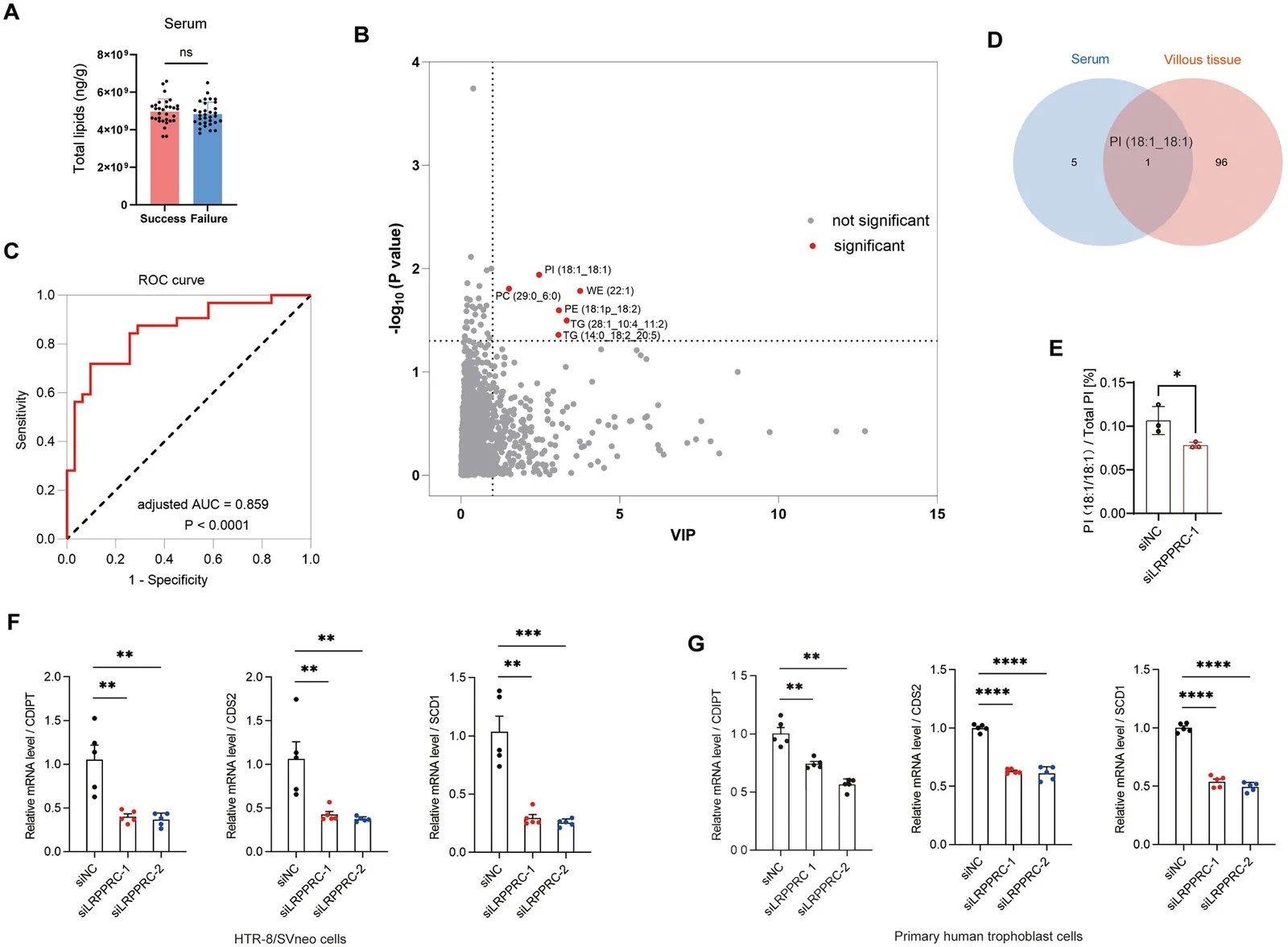
**Low preconception serum PI (18:1/18:1) levels predicted subsequent miscarriage in the RPL cohort.** (**A**) Total lipid levels in pre-gestational serum were compared between women with subsequent successful pregnancy (n = 32) and those with pregnancy failure (n = 31). (**B**) Major lipid subtypes in pre-gestational serum were compared between women with successful pregnancy outcomes and those with pregnancy failure. (**C**) ROC curve for a multivariate logistic regression model that integrates five lipid predictors (PI (18:1_18:1), PC (29:0_6:0), PE (18:1p_18:2), TG (28:1_10:4_11:2), WE (22:1)) in combination with a clinical covariate (the number of prior spontaneous pregnancy losses) to predict subsequent pregnancy outcomes. The corresponding AUC and *P*-value are indicated. (**D**) PI (18:1/18:1) decreases both in serum and villous tissues of RPL patients. (**E**) The proportion of PI (18:1/18:1)/Total PI decreases after the LRPPRC was knocked down in trophoblast cells. (**F**) RT–qPCR analysis of *CDIPT*, *CDS2*, and *SCD1* mRNA levels in HTR-8/SVneo cells. (**G**) RT–qPCR analysis of *CDIPT*, *CDS2*, and *SCD1* mRNA levels in primary trophoblast cells. *P < 0.05, **P < 0.01, ***P < 0.001, ****P < 0.0001. PI, phosphatidylinositol; RPL, recurrent pregnancy loss; ROC, receiver operating characteristic; PC, phosphatidylcholine; PE, phosphatidylethanolamine; TG, triglyceride; WE, wax ester; AUC, area under the curve; RT–qPCR, real-time quantitative polymerase chain reaction; *CDIPT*, CDP-diacylglycerol inositol 3-phosphatidyltransferase; *CDS2*, CDP-diacylglycerol synthase 2; *SCD1*, stearoyl-CoA desaturase 1; ns, not significant; VIP, variable importance in projection.

Strikingly, PI (18:1/18:1) was significantly reduced not only in RPL villous tissues but also in the preconception serum of patients who subsequently experienced pregnancy loss (Supplementary Table S5). Given the observed reduction of PI (18:1/18:1) in both RPL villous tissues and pre-gestational serum (Fig. 5D), we sought to determine whether LRPPRC directly regulates PI metabolism in trophoblasts. We performed LC-MS in HTR-8/SVneo cells following *LRPPRC* knockdown. The results confirmed that LRPPRC deficiency led to a significant decrease in the relative abundance of PI (18:1/18:1) among total PIs (Fig. 5E).

To provide direct regulatory evidence linking LRPPRC to the phospholipid biosynthetic pathway, we performed RT–qPCR analysis of key rate-limiting enzymes, including SCD1 (involved in fatty acid desaturation) as well as CDIPT and CDS2 (both involved in PI biosynthesis), in both LRPPRC-depleted HTR-8/SVneo cells and primary human trophoblasts. Our results demonstrated that knockdown of *LRPPRC* significantly reduced the mRNA levels of *SCD1*, *CDIPT*, and *CDS2* (Fig. 5F and G). Collectively, our findings suggest that PI plays a critical role in RPL pathogenesis, potentially by compromising trophoblast function, and highlight its promise as a predictive biomarker.

### Exogenous PI (18:1/18:1) rescued functional impairments in LRPPRC-deficient trophoblasts

To understand the importance of PI (18:1/18:1) as a regulator downstream of LRPPRC, we performed rescue experiments in trophoblast cells. For this purpose, LRPPRC-downregulated HTR-8/SVneo cells were treated with 50 μM PI (18:1/18:1). Functional assays demonstrated that PI (18:1/18:1) supplementation effectively restored cell proliferation (CCK-8 assay) and viability (ATP assay), while reducing cytotoxicity (LDH assay) (Fig. 6A and B). This restorative effect was further supported by a significant decrease in apoptosis, as measured by Annexin V/FITC staining (Fig. 6C). Consistent with these findings, western-blot analysis revealed downregulation of apoptosis-related proteins upon PI (18:1/18:1) treatment (Fig. 6D). Together, these results indicate that exogenous PI (18:1/18:1) supplementation can compensate for LRPPRC deficiency by ameliorating trophoblast dysfunction.

**Figure 6.**
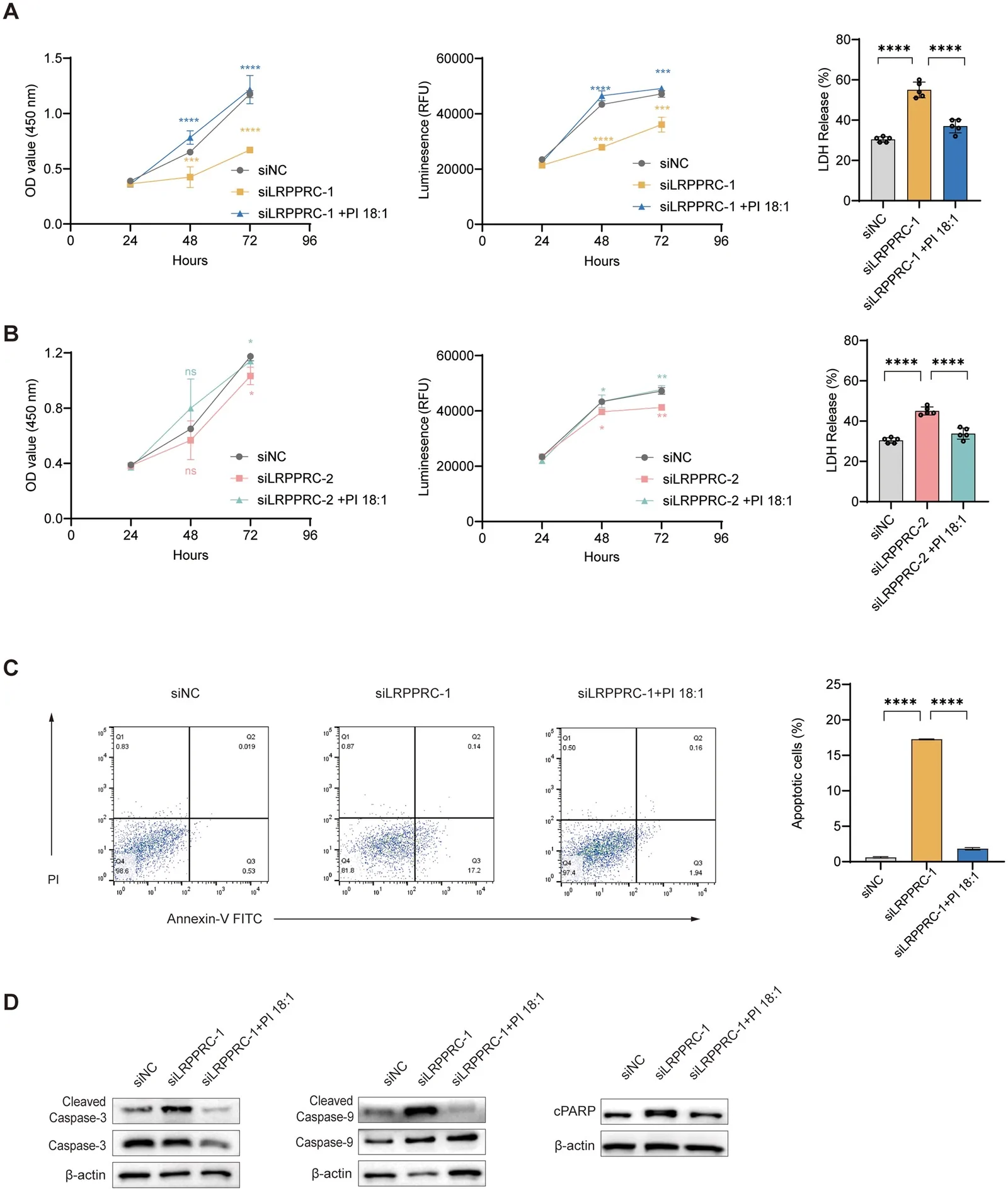
**Exogenous PI (18:1/18:1) supplementation rescued functional impairments in LRPPRC-deficient trophoblasts/**(**A and B**) Functional recovery after PI (18:1/18:1) supplementation (50 μM) in siLRPPRC-1 (A) and siLRPPRC-2 (B) transfected cells, assessed by CCK-8, ATP, and LDH assays. (**C**) Apoptosis analysis by Annexin V/propidium iodide flow cytometry (left) and corresponding quantitative results (right). (**D**) Western-blot analysis of caspase-3, caspase-9, and cPARP in cells under indicated treatments. *P < 0.05, **P < 0.01, ***P < 0.001, ****P < 0.0001. PI, phosphatidylinositol; LRPPRC, leucine-rich pentatricopeptide repeat-containing protein; CCK-8, Cell Counting Kit-8; ATP, adenosine 5′-triphosphate; LDH, lactate dehydrogenase; cPARP, cleaved poly (ADP-ribose) polymerase; OD, optical density; RFU, relative fluorescence units; siNC, negative control siRNA; ns, not significant; PI/FITC, propidium iodide/fluorescein isothiocyanate.

## Discussion

RPL represents a common yet frequently overlooked reproductive health issue, affecting ∼2.5% of women of reproductive age, particularly before 20–24 weeks of gestation (Dimitriadis *et al.*, 2020; Bender *et al.*, 2023). RPL not only imposes a physical burden but also has a profound impact on the psychological well-being of affected women. Approximately one-third of women attending specialized clinics for RPL are emotionally depressed, and one in five exhibit equivalent anxiety levels comparable to those of psychiatric outpatient populations (Rai and Regan, 2006). Meanwhile, the therapeutic options for RPL remain limited and highly individualized, underscoring the urgent need to elucidate its underlying mechanisms and develop novel treatment strategies.

In this study, we found LRPPRC, a recently identified m^6^A reader protein (Yu *et al.*, 2024), as a key differentially expressed regulator in RPL. Functional experiments revealed that LRPPRC silencing inhibited trophoblast viability and induced both apoptosis and oxidative stress. Mechanistically, transcriptomic analysis further indicated that LRPPRC deficiency significantly altered the expression of genes involved in phospholipid biosynthesis. Collectively, these findings establish LRPPRC as a critical maintainer of trophoblast function, potentially through its role in regulating lipid metabolism. Notably, these results were derived from a hypothesis-driven re-analysis of the GSE76862 dataset originally published by Li *et al.* (Tian *et al.*, 2016), who identified the YY1/MMP2 axis as a key regulator of trophoblast invasion in RPL. Our re-analysis was not intended as a comprehensive or definitive reinterpretation, but rather a focused examination of m^6^A-related and mitochondrial regulators based on emerging evidence linking these pathways to trophoblast function and RPL. This focused strategy identified *LRPPRC* as a candidate gene affecting trophoblast apoptosis and phospholipid metabolism, which are biological processes distinct from the invasion-related pathway reported by Li *et al.* Thus, our findings are complementary rather than contradictory to the original interpretation. And we also recognize that other dysregulated pathways may exist beyond the scope of this analysis.

LRPPRC has previously been well-established as a key regulator of energy metabolism in multiple cancers, which localizes at the outer nuclear and endoplasmic reticulum membrane, and interacts with mRNA *in vivo* (Tsuchiya *et al.*, 2004; Ge *et al.*, 2024). For instance, in ovarian cancer, it acts as an oncogene by enhancing glutaminolysis and the tricarboxylic acid cycle, thereby facilitating cancer cell survival and growth (Szulta *et al.*, 2025). Similarly, in urothelial carcinoma of the bladder, LRPPRC modulates ROS balance and protects cells from oxidative stress via mitochondrial mRNA metabolism (Wei *et al.*, 2021). These findings highlight LRPPRC’s function as a multifunctional mitochondrial protein that powers the high-energy requirements of rapidly dividing cells.

Our lipidomic analysis of villous tissues from RPL patients revealed a marked reduction in total lipids, including several phospholipid species. Moreover, we assessed the prognostic value of lipids and discovered that preconception serum levels of PI (18:1/18:1) were significantly lower in women with a history of RPL who went on to experience another miscarriage. Notably, supplementing LRPPRC-deficient cells with exogenous PI (18:1/18:1) effectively restored the proliferative deficit and attenuated the apoptosis. These findings suggest the potential contribution of PI (18:1/18:1) to RPL pathogenesis. PI (18:1/18:1) is a well-defined molecular species of PI and a lipokine with high bio-activity (Thurmer *et al.*, 2022), characterized by a glycerol backbone esterified with two oleic acid chains (18:1/18:1) and a myo-inositol headgroup. The biological activity of PI (18:1/18:1) can be understood from two complementary perspectives: its signaling functions via phosphoinositide derivatives and its biophysical effects on membrane properties. Regarding signaling, the inositol headgroup can be phosphorylated at multiple positions, yielding key signaling lipids such as phosphatidylinositol 4-phosphate (PI_4_P), phosphatidylinositol 4,5-bisphosphate (PIP_2_), and phosphatidylinositol 3,4,5-trisphosphate (PIP_3_). These phosphoinositides play an essential role in activating signaling cascades, including the PI3K/AKT/mTOR pathway, which governs cell survival, growth, and proliferation (Ai *et al.*, 2018; Cizmecioglu, 2022). Upon activation by growth factors or oncogenic signals, PI3K phosphorylates PIP_2_ to generate PIP_3_, which in turn recruits and activates AKT. Activated AKT then promotes cell cycle progression and suppresses apoptosis by phosphorylating downstream targets such as mTOR, FoxO, and Bad. Dysregulation of the PI3K/AKT pathway is frequently observed in cancer, where its hyperactivation drives uncontrolled proliferation and confers resistance to apoptosis (Ai *et al.*, 2018; Cizmecioglu, 2022). For instance, in hepatocellular carcinoma, knockdown of *PIK3R1* (a regulatory subunit of PI3K), significantly curtails cell proliferation and promotes apoptosis by downregulating p-PI3K, p-AKT, and p-mTOR (Ai *et al.*, 2018). Similarly, in acute myeloid leukemia, suppression of piRNA-32877, which interacts with the PI3K/AKT pathway, inhibits cell proliferation, and induces apoptosis via upregulation of caspase-3 and caspase-9 (Zeng *et al.*, 2020; Nasseri *et al.*, 2023). Beyond its signaling role, the specific fatty acyl composition of PI(18:1/18:1)—two oleic acid chains (18:1, cis-9)—critically influences membrane biophysical properties (Legaly *et al.*, 1977). Each cis double bond introduces a rigid kink into the hydrocarbon tail, which reduces acyl chain packing and increases membrane fluidity compared to fully saturated PI species (Li *et al.*, 1994; Jing *et al.*, 2004). This enhanced fluidity is functionally important at the maternal–fetal interface, where trophoblast cells undergo dynamic processes including migration, invasion, and syncytialization, all of which depend on proper membrane deformability and lateral mobility of membrane proteins such as integrins and matrix metalloproteinases (MMPs) (Mazzanti *et al.*, 1992; Spessotto *et al.*, 2006). Compared to polyunsaturated PI species (e.g. PI (20:4/20:4) containing arachidonic acid), PI (18:1/18:1) provides moderate fluidity without the high susceptibility to lipid peroxidation, as polyunsaturated chains contain bis-allylic hydrogens that readily generate reactive aldehydes under oxidative stress (Zhang *et al.*, 2023a), which is a known contributor to RPL pathogenesis (Ozkaya *et al.*, 2008). Thus, PI (18:1/18:1) may represent an optimal balance between membrane fluidity and oxidative stability. Its deficiency, as observed in RPL patients, could therefore compromise trophoblast membrane dynamics, thereby impairing invasive capacity and contributing to placental insufficiency. We will furtherly explore the exact mechanisms by which PI (18:1/18:1) affects trophoblast function, including both its signaling and membrane-remodeling roles.

Although *LRPPRC* knockdown disturbed the ratio of PI (18:1/18:1) in trophoblasts, the potential mechanisms remain unclear. PI (18:1/18:1) can be synthesized *de novo* from CDP-diacylglycerol via PI-synthase (e.g. CDIPT and CDS2) or modified from saturated precursors by SCD1 (Blunsom and Cockcroft, 2020); each route is governed by multiple rate-limiting steps that remain to be mapped. Given its role as an m^6^A reader, LRPPRC may post-transcriptionally fine-tune the expression of key enzymes regulating these metabolic routes. Our results demonstrated that knockdown of *LRPPRC* significantly reduced the mRNA levels of *SCD1*, *CDIPT*, and *CDS2*, supporting a regulatory role for LRPPRC in PI biosynthesis. Notably, *SCD1* is a well-characterized m^6^A target recognized by YTHDF1 (Meng *et al.*, 2024). Based on these findings, we hypothesize that LRPPRC may cooperate with canonical m^6^A readers such as YTHDF1 to recognize m^6^A sites on *SCD1* mRNA, thereby modulating PI metabolism. Future studies are warranted to test this possibility.

We acknowledge several limitations in this study. First, the analysis of preconception serum lipids was performed in a cohort of 63 women with a history of RPL. This sample size remains relatively modest, primarily due to the current availability of patients with documented follow-up pregnancy outcomes. Future studies with continued longitudinal follow-up in larger cohorts are warranted to validate our findings and improve predictive accuracy. Second, the precise molecular mechanism by which LRPPRC regulates PI-synthase or *SCD1* mRNA stability/translation in an m^6^A-dependent manner has not yet been fully elucidated. Further mechanistic investigations are required to dissect the regulatory role of LRPPRC in *SCD1* and other key enzymes involved in phospholipid metabolism. Third, our functional experiments were performed in cultured trophoblast cells, which may not fully capture the complexity of the human placental microenvironment. Future complementary validation using physiologically relevant human placental model systems, such as trophoblast organoids (Haider *et al.*, 2018; Turco *et al.*, 2018; Calvi and Turco, 2026), first‑trimester placental explants (Miller *et al.*, 2005; Zhao *et al.*, 2023), or maternal‑fetal interface assembloids (Shibata *et al.*, 2024), would further strengthen the mechanistic and translational significance of the proposed LRPPRC‑PI pathway.

In summary, LRPPRC deficiency contributed to RPL by impairing trophoblast function through the disruption of PI metabolism. Our findings underscore the promising role of PI (18:1/18:1) in maintaining trophoblast homeostasis and highlight its potential as a therapeutic target for RPL. Future studies should prioritize elucidating the precise molecular mechanisms by which this lipid species governs trophoblast behavior and evaluating its efficacy in mitigating placental disorders.

## Supplementary Material

deag123_Supplementary_Table_S1

deag123_Supplementary_Table_S2

deag123_Supplementary_Table_S3

deag123_Supplementary_Table_S4

deag123_Supplementary_Table_S5

## Data Availability

The data underlying this article are available in the article and in its Supplementary Material.

## References

[deag123-B1] Ai X , Xiang L, Huang Z, Zhou S, Zhang S, Zhang T, Jiang T. Overexpression of PIK3R1 promotes hepatocellular carcinoma progression. Biol Res 2018;51:52. 10.1186/s40659-018-0202-7 PMC6264640 30497511

[deag123-B2] Alvarez-Sanchez A , Grinat J, Doria-Borrell P, Mellado-Lopez M, Pedrera-Alcocer E, Malenchini M, Meseguer S, Hemberger M, Perez-Garcia V. The GPI-anchor biosynthesis pathway is critical for syncytiotrophoblast differentiation and placental development. Cell Mol Life Sci 2024;81:246. 10.1007/s00018-024-05284-2 PMC11143174 38819479

[deag123-B3] Bender AR , Christiansen OB, Elson J, Kolte AM, Lewis S, Middeldorp S, Mcheik S, Peramo B, Quenby S, Nielsen HS et al ESHRE guideline: recurrent pregnancy loss: an update in 2022. Hum Reprod Open 2023;2023:hoad002. 10.1093/hropen/hoad002 PMC9982362 36873081

[deag123-B4] Blunsom NJ , Cockcroft S. Phosphatidylinositol synthesis at the endoplasmic reticulum. Biochim Biophys Acta Mol Cell Biol Lipids 2020;1865:158471. 10.1016/j.bbalip.2019.05.015 31173893

[deag123-B5] Calvi I , Turco MY. Investigating human pregnancy with trophoblast organoids. FEBS Lett 2026. 10.1002/1873-3468.70344 42028669

[deag123-B6] Cizmecioglu O. p110alpha activity at the M-to-G1 transition is critical for cellular proliferation and reentry into the cell cycle. Turk J Biol 2022;46:207–215. 10.55730/1300-0152.2609 PMC10388040 37529255

[deag123-B7] Dimitriadis E , Menkhorst E, Saito S, Kutteh WH, Brosens JJ. Recurrent pregnancy loss. Nat Rev Dis Primers 2020;6:98. 10.1038/s41572-020-00228-z 33303732

[deag123-B8] Ge Y , Janson V, Liu H. Comprehensive review on leucine-rich pentatricopeptide repeat-containing protein (LRPPRC, PPR protein): a burgeoning target for cancer therapy. Int J Biol Macromol 2024;282:136820. 10.1016/j.ijbiomac.2024.136820 39476900

[deag123-B9] Gelinas R , Levesque C, Thompson Legault J, Rivard ME, Villeneuve L, Laprise C, Rioux JD. Human induced pluripotent stem cells (hiPSCs) derived cells reflect tissue specificity found in patients with Leigh syndrome French Canadian variant (LSFC). Front Genet 2024;15:1375467. 10.3389/fgene.2024.1375467 PMC11066297 38706791

[deag123-B10] Haider S , Meinhardt G, Saleh L, Kunihs V, Gamperl M, Kaindl U, Ellinger A, Burkard TR, Fiala C, Pollheimer J et al Self-renewing trophoblast organoids recapitulate the developmental program of the early human placenta. Stem Cell Reports 2018;11:537–551. 10.1016/j.stemcr.2018.07.004 PMC6092984 30078556

[deag123-B11] Jiang Y , Du M, Wu M, Zhu Y, Zhao X, Cao X, Li X, Long P, Li W, Hu B. Phosphatidic acid improves reprogramming to pluripotency by reducing apoptosis. Stem Cells Dev 2016;25:43–54. 10.1089/scd.2015.0159 26451619

[deag123-B12] Jing B , Tokutake N, McCullough DH III, Regen SL. A quantitative assessment of the influence of permanent kinks on the mixing behavior of phospholipids in cholesterol-rich bilayers. J Am Chem Soc 2004;126:15344–15345. 10.1021/ja044517c 15563137

[deag123-B13] Legaly G , Weiss A, Stuke E. Effect of double-bonds on bimolecular films in membrane models. Biochim Biophys Acta 1977;470:331–341. 10.1016/0005-2736(77)90125-0 921958

[deag123-B14] Lehenauer T , Jaksch-Bogensperger H, Huber S, Weghuber D, Fischer T, Mayr JA, Kofler B, Neumayer B, Gharehbaghi D, Duggan-Peer M et al Mitochondrial oxidative phosphorylation alterations in placental tissues from early- and late-onset preeclampsia. Int J Mol Sci 2025;26:3951. 10.3390/ijms26093951 PMC12071294 40362193

[deag123-B15] Li S , Lin HN, Wang ZQ, Huang C. Identification and characterization of kink motifs in 1-palmitoyl-2-oleoyl-phosphatidylcholines: a molecular mechanics study. Biophys J 1994;66:2005–2018. 10.1016/S0006-3495(94)80993-2 PMC1275925 8075334

[deag123-B16] Li S , Liu H, Ruan Z, Guo R, Sun C, Tang Y, Huang X, Gao T, Hao S, Li H et al Landscape analysis of m6A modification regulators related biological functions and immune characteristics in myasthenia gravis. J Transl Med 2023;21:166. 10.1186/s12967-023-03947-5 PMC9983271 36864526

[deag123-B17] Li XC , Jin F, Wang BY, Yin XJ, Hong W, Tian FJ. The m6A demethylase ALKBH5 controls trophoblast invasion at the maternal-fetal interface by regulating the stability of CYR61 mRNA. Theranostics 2019;9:3853–3865. 10.7150/thno.31868 PMC6587351 31281518

[deag123-B18] Liu H , Zheng J, Liao A. The regulation and potential roles of m6A modifications in early embryonic development and immune tolerance at the maternal-fetal interface. Front Immunol 2022;13:988130. 10.3389/fimmu.2022.988130 PMC9549360 36225914

[deag123-B19] Liu W-W , Zheng S-Q, Li T, Fei Y-F, Wang C, Zhang S, Wang F, Jiang G-M, Wang H. RNA modifications in cellular metabolism: implications for metabolism-targeted therapy and immunotherapy. Signal Transduct Target Ther 2024;9:70. 10.1038/s41392-024-01777-5 PMC10966055 38531882

[deag123-B20] Mazzanti L , Rabini RA, Biagini G, Pugnaloni A, de Pirro R, Faloia E, Mancini V, Romanini C, Cester N. Changes in membrane fluidity and Na+/K(+)-ATPase activity during human trophoblast cell culture. Eur J Biochem 1992;206:881–885. 10.1111/j.1432-1033.1992.tb16996.x 1318838

[deag123-B21] McKeegan PJ , Sturmey RG. The role of fatty acids in oocyte and early embryo development. Reprod Fertil Dev 2011;24:59–67. 10.1071/RD11907 22394718

[deag123-B22] Meng J , Yan C, Liu J. LDHA-mediated histone lactylation promotes the nonalcoholic fatty liver disease progression through targeting the METTL3/ YTHDF1/SCD1 m6A axis. Physiol Res 2024;73:985–999. 10.33549/physiolres.935289 PMC11835221 39903889

[deag123-B23] Meyer KD , Saletore Y, Zumbo P, Elemento O, Mason CE, Jaffrey SR. Comprehensive analysis of mRNA methylation reveals enrichment in 3′ UTRs and near stop codons. Cell 2012;149:1635–1646. 10.1016/j.cell.2012.05.003 PMC3383396 22608085

[deag123-B24] Miller RK , Genbacev O, Turner MA, Aplin JD, Caniggia I, Huppertz B. Human placental explants in culture: approaches and assessments. Placenta 2005;26:439–448. 10.1016/j.placenta.2004.10.002 15950058

[deag123-B25] Nasseri S , Sharifi M, Mehrzad V. Effects of hsa-piR-32877 suppression with antisense LNA GapmeRs on the proliferation and apoptosis of human acute myeloid leukemia cells. Int J Mol Cell Med 2023;12:18–29. 10.22088/IJMCM.BUMS.12.1.18 PMC10629728 37942262

[deag123-B26] Ozkaya O , Sezik M, Kaya H. Serum malondialdehyde, erythrocyte glutathione peroxidase, and erythrocyte superoxide dismutase levels in women with early spontaneous abortions accompanied by vaginal bleeding. Med Sci Monit 2008;14:CR47–CR51. 18160945

[deag123-B27] Porcellati G , Curti B, Luciani S. Phospholipid phosphoric esters in the human placenta. Nature 1959;184:1870–1871. 10.1038/1841870a0 14434213

[deag123-B28] Powell TL , Ferchaud-Roucher V, Madi L, Uhlson C, Zemski-Berry K, Kramer AC, Erickson K, Palmer C, Chassen SS, Castillo-Castrejon M. Synthesis of phospholipids in human placenta. Placenta 2024;147:12–20. 10.1016/j.placenta.2024.01.007 PMC10923060 38278000

[deag123-B29] Rai R , Regan L. Recurrent miscarriage. Lancet 2006;368:601–611. 10.1016/S0140-6736(06)69204-0 16905025

[deag123-B30] Ritchie ME , Phipson B, Wu D, Hu Y, Law CW, Shi W, Smyth GK. limma powers differential expression analyses for RNA-sequencing and microarray studies. Nucleic Acids Res 2015;43:e47. 10.1093/nar/gkv007 PMC4402510 25605792

[deag123-B31] Romero R , Kusanovic JP, Chaiworapongsa T, Hassan SS. Placental bed disorders in preterm labor, preterm PROM, spontaneous abortion and abruptio placentae. Best Pract Res Clin Obstet Gynaecol 2011;25:313–327. 10.1016/j.bpobgyn.2011.02.006 PMC3092823 21388889

[deag123-B32] Sah N , Thornburg K, Slamecka J, Soncin F. Metabolism and homeostasis of energy in trophoblast cells of the placenta: from development to disease. Biol Reprod 2026;114:497–510. 10.1093/biolre/ioaf273 PMC13471717 41358913

[deag123-B33] Sasarman F , Brunel-Guitton C, Antonicka H, Wai T, Shoubridge EA; LSFC Consortium. LRPPRC and SLIRP interact in a ribonucleoprotein complex that regulates posttranscriptional gene expression in mitochondria. Mol Biol Cell 2010;21:1315–1323. 10.1091/mbc.E10-01-0047 PMC2854090 20200222

[deag123-B34] Shibata S , Endo S, Nagai LAE, E HK, Oike A, Kobayashi N, Kitamura A, Hori T, Nashimoto Y, Nakato R et al Modeling embryo-endometrial interface recapitulating human embryo implantation. Sci Adv 2024;10:eadi4819. 10.1126/sciadv.adi4819 PMC10889356 38394208

[deag123-B35] Spessotto P , Bulla R, Danussi C, Radillo O, Cervi M, Monami G, Bossi F, Tedesco F, Doliana R, Colombatti A. EMILIN1 represents a major stromal element determining human trophoblast invasion of the uterine wall. J Cell Sci 2006;119:4574–4584. 10.1242/jcs.03232 17074837

[deag123-B36] Szulta A , Wang L, Hasan A, Kinter M, Pranay A, Zhu J, Humphries KM, Loveland B, Griffin TM, Bieniasz M. The identification of a key regulator of mitochondrial metabolism, the LRPPRC protein, as a novel therapeutic target in SDHA-overexpressing ovarian tumors. Cancers (Basel) 2025;17:1942. 10.3390/cancers17121942 PMC12190274 40563592

[deag123-B37] Thurmer M , Gollowitzer A, Pein H, Neukirch K, Gelmez E, Waltl L, Wielsch N, Winkler R, Loser K, Grander J et al PI(18:1/18:1) is a SCD1-derived lipokine that limits stress signaling. Nat Commun 2022;13:2982. 10.1038/s41467-022-30374-9 PMC9142606 35624087

[deag123-B38] Tian FJ , Cheng YX, Li XC, Wang F, Qin CM, Ma XL, Yang J, Lin Y. The YY1/MMP2 axis promotes trophoblast invasion at the maternal-fetal interface. J Pathol 2016;239:36–47. 10.1002/path.4694 PMC5071713 27071480

[deag123-B39] Tsuchiya N , Fukuda H, Nakashima K, Nagao M, Sugimura T, Nakagama H. LRP130, a single-stranded DNA/RNA-binding protein, localizes at the outer nuclear and endoplasmic reticulum membrane, and interacts with mRNA in vivo. Biochem Biophys Res Commun 2004;317:736–743. 10.1016/j.bbrc.2004.03.103 15081402

[deag123-B40] Turco MY , Gardner L, Kay RG, Hamilton RS, Prater M, Hollinshead MS, McWhinnie A, Esposito L, Fernando R, Skelton H et al Trophoblast organoids as a model for maternal-fetal interactions during human placentation. Nature 2018;564:263–267. 10.1038/s41586-018-0753-3 PMC7220805 30487605

[deag123-B41] Wang XH , Xu S, Zhou XY, Zhao R, Lin Y, Cao J, Zang WD, Tao H, Xu W, Li MQ et al Low chorionic villous succinate accumulation associates with recurrent spontaneous abortion risk. Nat Commun 2021;12:3428. 10.1038/s41467-021-23827-0 PMC8187647 34103526

[deag123-B42] Wei W-S , Wang N, Deng M-H, Dong P, Liu J-Y, Xiang Z, Li X-D, Li Z-Y, Liu Z-H, Peng Y-L et al LRPPRC regulates redox homeostasis via the circANKHD1/FOXM1 axis to enhance bladder urothelial carcinoma tumorigenesis. Redox Biol 2021;48:102201. 10.1016/j.redox.2021.102201 PMC8645923 34864630

[deag123-B43] Xu F , Morin C, Mitchell G, Ackerley C, Robinson BH. The role of the LRPPRC (leucine-rich pentatricopeptide repeat cassette) gene in cytochrome oxidase assembly: mutation causes lowered levels of COX (cytochrome c oxidase) I and COX III mRNA. Biochem J 2004;382:331–336. 10.1042/BJ20040469 PMC1133946 15139850

[deag123-B44] Xue Q , Wang W, Liu J, Wang D, Zhang T, Shen T, Liu X, Wang X, Shao X, Zhou W et al LRPPRC confers enhanced oxidative phosphorylation metabolism in triple-negative breast cancer and represents a therapeutic target. J Transl Med 2025;23:372. 10.1186/s12967-024-05946-6 PMC11938637 40133967

[deag123-B45] Ye Q , Zeng X, Cai S, Qiao S, Zeng X. Mechanisms of lipid metabolism in uterine receptivity and embryo development. Trends Endocrinol Metab 2021;32:1015–1030. 10.1016/j.tem.2021.09.002 34625374

[deag123-B46] Yu Y , Deng H, Wang W, Xiao S, Zheng R, Lv L, Wang H, Chen J, Zhang B. LRPPRC promotes glycolysis by stabilising LDHA mRNA and its knockdown plus glutamine inhibitor induces synthetic lethality via m(6) A modification in triple-negative breast cancer. Clin Transl Med 2024;14:e1583. 10.1002/ctm2.1583 PMC10875709 38372449

[deag123-B47] Zeng Q , Wan H, Zhao S, Xu H, Tang T, Oware KA, Qu S. Role of PIWI-interacting RNAs on cell survival: proliferation, apoptosis, and cycle. IUBMB Life 2020;72:1870–1878. 10.1002/iub.2332 32697419

[deag123-B48] Zhang X , Hou L, Guo Z, Wang G, Xu J, Zheng Z, Sun K, Guo F. Lipid peroxidation in osteoarthritis: focusing on 4-hydroxynonenal, malondialdehyde, and ferroptosis. Cell Death Discov 2023a;9:320. 10.1038/s41420-023-01613-9 PMC10465515 37644030

[deag123-B49] Zhang M , Huang N, Gao Y, Feng Z, Kang B, Guo H, Jiang J, Liao S, Wang H. HNRNPC mediated m(6)A methylation of 5-methyltetrahydrofolate-homocysteine methyltransferase and involved in the occurrence of RSA. J Reprod Immunol 2023b;160:104160. 10.1016/j.jri.2023.104160 37857158

[deag123-B50] Zhang L , Zhao J, Lam SM, Chen L, Gao Y, Wang W, Xu Y, Tan T, Yu H, Zhang M et al Low-input lipidomics reveals lipid metabolism remodelling during early mammalian embryo development. Nat Cell Biol 2024;26:278–293. 10.1038/s41556-023-01341-3 38302721

[deag123-B51] Zhao X , Zhang Z, Zhu Q, Luo Y, Ye Q, Shi S, He X, Zhu J, Zhang D, Xia W et al Modeling human ectopic pregnancies with trophoblast and vascular organoids. Cell Rep 2023;42:112546. 10.1016/j.celrep.2023.112546 37224015

