# Supplementary material for: LRPPRC deficiency impairs trophoblast function through disrupted phosphatidylinositol metabolism in recurrent pregnancy loss
Source: Hum Reprod. 2026 Jul 28;41(11):1909–22. doi: 10.1093/humrep/deag123 (PMC13726757; doi:10.1093/humrep/deag123)
Supplement: Supplementary file 1 [file deag123_supplementary_table_s1.pdf]

Human Reproduction, 2026, 1–1
https://doi.org/10.1093/humrep/deag123
Supplementary Data

human reproduction
OXFORD

Supplementary Table S1. Characteristics of villous tissue donors.

Variables	NOR (n = 11)[a]	RPL (n = 11)[b]	*P*-value[c]
Age (years) (mean±SD)	29.45 ± 4.18	32.36 ± 4.30	0.123
BMI (kg/m^2) (mean±SD)	19.50 ± 1.17	21.76 ± 2.75	0.025
Number of spontaneous pregnancy losses (median (q_1, q_3))	0.00 (0.00, 0.00)	3.00 (2.00, 3.00)	<0.001

[a] NOR villous tissue donors were randomly selected from women with a clinically normal pregnancy history undergoing legal termination for non-medical reasons.
[b] RPL villous tissue donors were selected from patients with recurrent pregnancy loss undergoing dilation and curettage for embryonic arrest, and had normal chorionic villus karyotype results from clinical testing.
[c] *P*-values were calculated using a two-tailed unpaired Student's *t*-test for normally distributed data (age), or a two-tailed unpaired *t*-test with Welch's correction for unequal variances (BMI), and a two-tailed Mann–Whitney test for non-normally distributed data (number of spontaneous pregnancy losses).
q_1, first quartile; q_3, third quartile; NOR, normal; RPL, recurrent pregnancy loss.
