# Supplementary material for: LRPPRC deficiency impairs trophoblast function through disrupted phosphatidylinositol metabolism in recurrent pregnancy loss
Source: Hum Reprod. 2026 Jul 28;41(11):1909–22. doi: 10.1093/humrep/deag123 (PMC13726757; doi:10.1093/humrep/deag123)
Supplement: Supplementary file 2 [file deag123_supplementary_table_s2.pdf]

Human Reproduction, 2026, 1–1
https://doi.org/10.1093/humrep/deag123
Supplementary Data

human reproduction
OXFORD

Supplementary Table S2. Primer sequences used in this study.

Primer name	Sequences (5′-3′)	Roles
ACTB-F	CCATTGGCAATGAGCGGTTCC	qPCR
ACTB-R	CGGATGTCCACGTCACACTTCA	qPCR
LRPPRC-F	GCTCATAGGATATGGGACACACT	qPCR
LRPPRC-R	CCAGGAAATCAGTTGGTGAGAAT	qPCR
CDIPT-F	AGCATGAGCTTGGACGTGGC	qPCR
CDIPT-R	GGTGAACAGAGCAGGCCTAGAG	qPCR
CDS2-F	ATGGATCGCTTTGACTGCCA	qPCR
CDS2-R	CTTGGGTTAGGGCCTCTGAT	qPCR
SCD1-F	TTCCCGACGTGGCTTTTTCT	qPCR
SCD1-R	AGCCAGGTTTGTAGTACCTCC	qPCR
siLRPPRC-1	GATGTCCTAAAGCAACTCATA	siRNA
siLRPPRC-2	ATGATAAAGTAGAAGATGCCT	siRNA
Negative control	UUCUCCGAACGUGUCACGU	siRNA

F, forward primer; R, reverse primer; siRNA, small interfering RNA; qPCR, quantitative polymerase chain reaction; ACTB, β-Actin; LRPPRC, leucine-rich pentatricopeptide repeat containing; CDIPT, CDP-diacylglycerol inositol 3-phosphatidyltransferase; CDS2, CDP-diacylglycerol synthase 2; SCD1, stearoyl-CoA desaturase 1.
