# Supplementary material for: LRPPRC deficiency impairs trophoblast function through disrupted phosphatidylinositol metabolism in recurrent pregnancy loss
Source: Hum Reprod. 2026 Jul 28;41(11):1909–22. doi: 10.1093/humrep/deag123 (PMC13726757; doi:10.1093/humrep/deag123)
Supplement: Supplementary file 3 [file deag123_supplementary_table_s3.pdf]

Human Reproduction, 2026, 1–1
https://doi.org/10.1093/humrep/deag123
Supplementary Data

human reproduction OXFORD

Supplementary Table S3. Expression profiles of 23 m_6A regulators in the GSE76862 dataset.

Gene name	Log_2FoldChange	FoldChange	*P*-value
ALKBH5	0.2257	1.1693	0.2704
CBLL1	−0.1039	0.9305	0.4727
ELAVL1	−0.0437	0.9702	0.7943
FMR1	−1.0578	0.4804	0.1746
FTO	0.2115	1.1579	0.2622
HNRNPA2B1	−0.4057	0.7549	0.0485
HNRNPC	−0.6749	0.6264	0.0088
IGF2BP1	−1.8687	0.2738	0.1049
IGF2BP2	0.0080	1.0055	0.9941
IGF2BP3	−2.4924	0.1777	0.1822
LRPPRC[a]	−1.1631	0.4466	0.0201
METTL14	0.0399	1.0281	0.8939
METTL3	−0.2652	0.8321	0.2456
RBM15	−0.1940	0.8742	0.3537
RBM15B	−0.0722	0.9512	0.5467
WTAP	0.0321	1.0225	0.8169
YTHDC1	−0.2243	0.8560	0.5068
YTHDC2	0.2241	1.1681	0.1123
YTHDF1	−0.0521	0.9645	0.8061
YTHDF2	−0.4124	0.7514	0.1392
YTHDF3	−0.3092	0.8071	0.1137
ZC3H13	0.0110	1.0077	0.9625
VIRMA	−0.4898	0.7121	0.0154

[a] Identified based on an uncorrected threshold of $P < 0.05$ and $|\log_2\text{FoldChange}| > 1$. ALKBH5, AlkB homolog H5; CBLL1, Cbl Proto-Oncogene Like 1; ELAVL1, ELAV like RNA binding protein 1; FMR1, fragile X mental retardation 1; FTO, fat mass and obesity-associated protein; HNRNPA2B1, heterogeneous nuclear ribonucleoprotein A2/B1; HNRNPC, heterogeneous nuclear ribonucleoprotein C; IGF2BP1, insulin-like growth factor 2 mRNA-binding proteins 1; IGF2BP2, insulin-like growth factor 2 mRNA-binding proteins 2; IGF2BP3, insulin-like growth factor 2 mRNA-binding proteins 3; LRPPRC, leucine-rich pentatricopeptide repeat containing; METTL14, methyltransferase-like 14; METTL3, methyltransferase-like 3; RBM15, RNA binding motif protein 15; RBM15B, RNA binding motif protein 15B; WTAP, Wilms tumor 1-associating protein; YTHDC1, YTH N6-methyladenosine RNA binding protein C1; YTHDC2, YTH N6-methyladenosine RNA binding protein C2; YTHDF1, YTH N6-methyladenosine RNA binding protein F1; YTHDF2, YTH N6-methyladenosine RNA binding protein F2; YTHDF3, YTH N6-methyladenosine RNA binding protein F3; ZC3H13, zinc finger CCCH-type containing 13; VIRMA, Vir-like m6A methyltransferase-associated protein.
