# Supplementary material for: LRPPRC deficiency impairs trophoblast function through disrupted phosphatidylinositol metabolism in recurrent pregnancy loss
Source: Hum Reprod. 2026 Jul 28;41(11):1909–22. doi: 10.1093/humrep/deag123 (PMC13726757; doi:10.1093/humrep/deag123)
Supplement: Supplementary file 4 [file deag123_supplementary_table_s4.pdf]

Human Reproduction, 2026, 1–1
https://doi.org/10.1093/humrep/deag123
Supplementary Data

human reproduction
OXFORD

Supplementary Table S4. Diagnostic performance of candidate lipid biomarkers and clinical predictors.

Predictor	AUC	95% CI	*P*-value	Sensitivity	Specificity	Included in final model
PI (18:1_18:1)	0.693	0.562–0.823	**0.0086**[b]	0.656	0.677	Yes
PC (29:0_6:0)	0.645	0.502–0.788	**0.0477**[b]	0.438	1.000	Yes
PE (18:1p_18:2)	0.635	0.496–0.774	0.0654	0.406	0.871	Yes
TG (28:1_10:4_11:2)	0.680	0.545–0.816	**0.0139**[b]	0.938	0.484	Yes
TG (14:0_18:2_20:5)	0.606	0.459–0.753	0.1489	0.562	0.806	No
WE (22:1)	0.659	0.519–0.799	**0.0298**[b]	0.625	0.774	Yes
Number of prior spontaneous pregnancy losses	0.637	0.514–0.759	0.0625	0.719	0.548	Yes
Age	0.551	0.407–0.696	0.4832	0.531	0.613	No
BMI	0.552	0.407–0.698	0.4747	0.562	0.613	No
Five included lipids (unadjusted)	0.844	0.747–0.941	**<0.0001**[b]	0.656	0.903	Not available
Five included lipids (adjusted)	0.859[a]	0.767–0.951	**<0.0001**[b]	0.719	0.903	Not available

[a] AUC value for the five included lipids was adjusted for the covariate of the number of prior spontaneous pregnancy losses.
[b] Bold values indicate statistical significance at the threshold of $P < 0.05$.
PI, phosphatidylinositol; PC, phosphatidylcholine; PE, phosphatidylethanolamine; TG, triglyceride; WE, wax ester; AUC, area under the curve.

© The Author(s) 2026. Published by Oxford University Press on behalf of European Society of Human Reproduction and Embryology.
This is an Open Access article distributed under the terms of the Creative Commons Attribution License (https://creativecommons.org/licenses/by/4.0/), which permits unrestricted reuse, distribution, and reproduction in any medium, provided the original work is properly cited.
Human Reproduction, 2026, 00, 1–14
https://doi.org/10.1093/humrep/deag123
Original Article
